# *Dentinogenesis Imperfecta* in Primary Dentition: Case Report

**DOI:** 10.3390/reports9020115

**Published:** 2026-04-10

**Authors:** Līna Petrova, Jūlija Ustiča, Ingrīda Čēma

**Affiliations:** 1 Faculty of Dentistry, Rīga Stradiņš University, LV-1007 Riga, Latvia; 2 Dental Clinic “Zobu Feja”, LV-1011 Riga, Latvia; info@zobufeja.lv; 3 Department of Maxillo-Facial Surgery and Oral Medicine, Rīga Stradiņš University, LV-1007 Riga, Latvia; ingrida.cema@rsu.lv

**Keywords:** *dentinogenesis imperfecta*, primary dentition, pediatric dentistry, tooth abnormalities

## Abstract

**Background and Clinical Significance:** *Dentinogenesis imperfecta* is a hereditary dentin disorder that compromises tooth structure, esthetics, and function. **Case Presentation:** We report the case of a 1.5-year-old female presenting with generalized discoloration of the primary dentition and intermittent sensitivity to thermal stimuli. The diagnosis of *dentinogenesis imperfecta* was established based on characteristic clinical features, radiographic findings, and a positive family history. The patient was followed longitudinally from 2020 to 2025, with documentation of diagnostic findings, radiographic changes, therapeutic interventions, and outcomes. Management included placement of composite veneers on the maxillary incisors for esthetic rehabilitation and sealants on second primary molars as a preventive measure. Although various management approaches have been described in the literature, evidence regarding optimal strategies and long-term outcomes in the primary dentition remains limited. This case highlights the occurrence of asymptomatic periapical pathology and root resorption despite minimal clinical symptoms, underscoring the challenges of relying on symptom-based assessment alone. **Conclusions:** Early diagnosis, regular radiographic monitoring, and individualized, risk-based treatment planning are essential in managing *dentinogenesis imperfecta*. This case emphasizes the importance of recognizing asymptomatic disease progression and integrating psychosocial considerations into comprehensive care.

## 1. Introduction and Clinical Significance

*Dentinogenesis imperfecta* (DI) is a hereditary disorder affecting dentin development, with a reported incidence of approximately 1:6000–1:8000 individuals [1]. It follows an autosomal dominant inheritance pattern, with a 50% probability of transmission when one parent is affected [2], and may occur as an isolated dental condition or in association with *osteogenesis imperfecta* (OI) [1,3]. Differential diagnoses include dentin dysplasia, hypocalcified forms of *amelogenesis imperfecta*, and other conditions associated with tooth discoloration or structural abnormalities [2].

The condition was first described in the late 19th and early 20th centuries, and the term “hereditary opalescent dentin” was introduced by Hodge et al. (1936) [4], followed by “*dentinogenesis imperfecta*” proposed by Robert and Schour [5]. DI is inherited with high penetrance and a low mutation rate [1,5].

According to the classification proposed by Shields (1973) [6], DI is divided into three types: Type I, associated with OI; Type II, occurring without systemic involvement; and Type III, a rare variant characterized by enlarged pulp chambers (“shell teeth”) [7,8,9]. Although Types I and II are genetically distinct, they demonstrate similar clinical and radiographic features [10].

Histologically, DI is characterized by abnormal dentin structure, including irregular tubules and areas of unmineralized matrix [3,10,11]. Mutations in the dentin sialophosphoprotein (DSPP) gene are associated with DI types II and III and affect dentin mineralization and repair processes [12,13].

Clinically, DI presents with generalized tooth discoloration (amber, gray, or brown shades), increased susceptibility to enamel fracture, and accelerated dentin wear [14,15]. Enamel fracture or detachment exposes the underlying dentin, leading to accelerated wear and increased susceptibility to dental caries [15,16]. The exposed, hypomineralized dentin undergoes accelerated attrition, which may range from minor erosive facets to complete loss of the crown structure [17]. Primary teeth are generally more severely affected than the permanent dentition [11]. Radiographically, affected teeth typically show bulbous crowns, cervical constriction, shortened roots, and partial or complete obliteration of the pulp chambers, with possible periapical radiolucencies even in the absence of clinical symptoms [7,16,18,19].

These features reflect an underlying dentin defect rather than primary enamel pathology [10,16].

Management in children aims to preserve tooth vitality, maintain function and occlusal stability, and address esthetic and psychosocial concerns [5]. Treatment approaches are individualized and may include preventive strategies, adhesive restorations, or full-coverage crowns depending on disease severity and patient-related factors [19,20]. However, evidence regarding optimal management and long-term outcomes in the primary dentition remains limited.

To the best of the authors’ knowledge, there are no published reports specifically describing *dentinogenesis imperfecta* in patients from the Baltic region. This case report presents the longitudinal clinical course of a young patient with DI in the primary dentition, with emphasis on asymptomatic radiographic pathology and its implications for monitoring and treatment planning.

## 2. Case Presentation

Written informed consent was obtained from the patient’s parents prior to the preparation of this case report. The study was conducted in accordance with the principles of the Declaration of Helsinki and was approved by the Ethics Committee of Rīga Stradiņš University (Decision No. 2-PĒK-4/1272/2025, 2 December 2025).

Standardized intraoral photographs and periapical radiographs were obtained (Canon (Tokyo, Japan), Belmont (Japan)) and analyzed with dedicated imaging software.

A 1.5-year-old girl with no known systemic disease presented for her first dental visit in May 2020. Clinical examination revealed generalized discoloration of the primary dentition, with crowns exhibiting brown–blue hues, suggestive of a hereditary defect of dentin formation. The clinical presentation—characterized by opalescent discoloration affecting all erupted primary teeth—together with a positive family history of similarly affected primary dentition without involvement of permanent teeth, supported a provisional diagnosis of *dentinogenesis imperfecta* (DI).

Differential diagnoses included *amelogenesis imperfecta* (AI), *osteogenesis imperfecta* (OI), and medication-induced tooth discoloration. AI was considered less likely due to the absence of primary enamel defects, such as hypoplasia or hypomineralization, and the presence of dentin-related discoloration rather than isolated enamel involvement. OI was not clinically supported at the time of examination, as no history or signs of bone fragility, recurrent fractures, blue sclerae, or growth abnormalities were reported. Medication-induced discoloration was excluded based on the absence of relevant medical history or exposure.

Although genetic consultation was recommended, it was not pursued, as the patient’s family declined further evaluation. The diagnosis was therefore established on clinical and familial grounds, with continued follow-up planned to monitor for any emerging features that might warrant further systemic or genetic evaluation. A detailed family pedigree was not obtained, as the assessment of family history was based on parental reporting rather than a structured genealogical analysis. Therefore, although the reported pattern is suggestive of autosomal dominant inheritance, this could not be formally confirmed within the scope of this case report. The parents reported intermittent sensitivity to thermal stimuli during eating, but no spontaneous pain, swelling, or functional impairment. The child was otherwise in good general health. Family history revealed that several relatives had exhibited similar discoloration of primary teeth, described as “brown teeth,” without involvement of the permanent dentition, further supporting a hereditary dental condition. The patient was enrolled in a structured recall program and attended follow-up visits in December 2020, February 2021, April 2021, April 2022, and August 2022.

**Figure 1:**
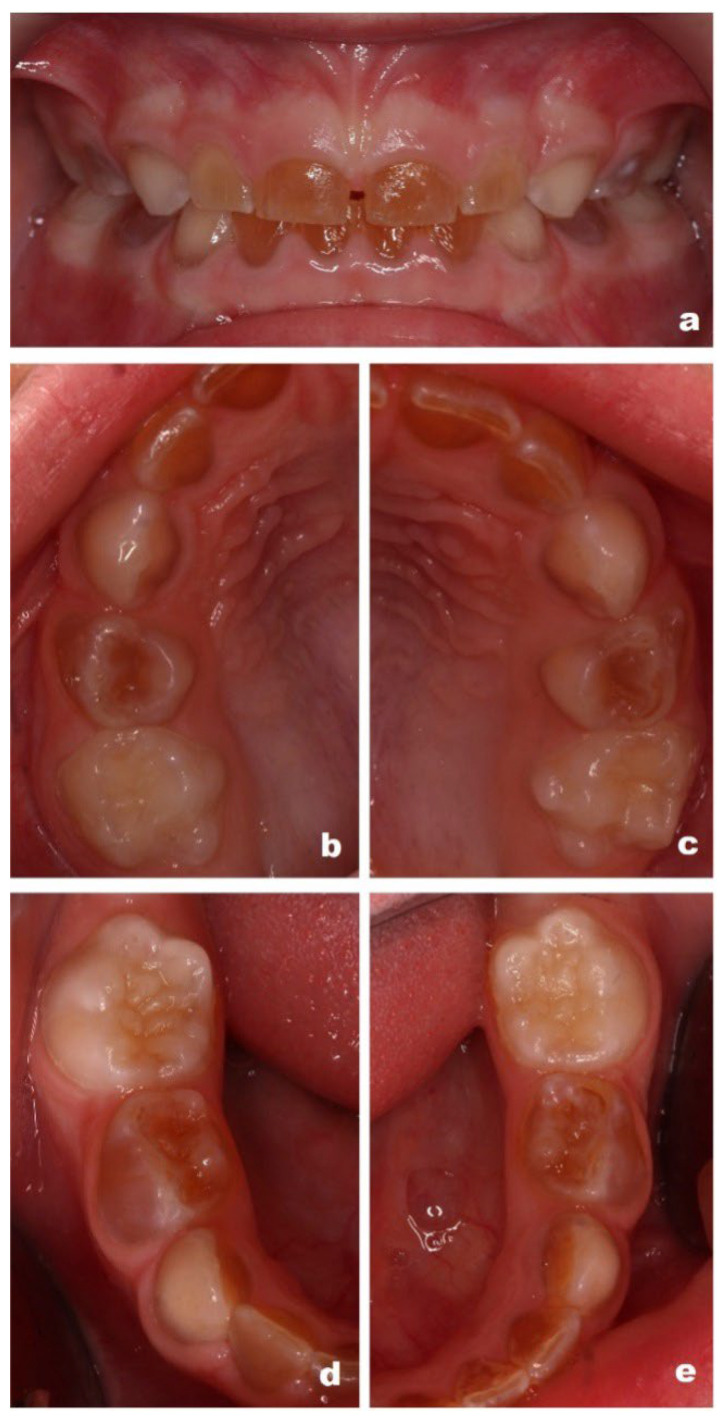
Intraoral photographs obtained in September 2022 (patient aged 3 years), demonstrating generalized discoloration of the primary dentition consistent with DI: (**a**) anterior teeth in occlusion; (**b**) upper right quadrant; (**c**) upper left quadrant; (**d**) lower right quadrant; (**e**) lower left quadrant.

Given the known risk of asymptomatic pulpal and periapical pathology in DI, intraoral photographs and periapical radiographs were obtained in September 2022 (Figure 1a–e) as part of a preventive and diagnostic assessment, despite the absence of acute clinical complaints.

**Figure 2:**
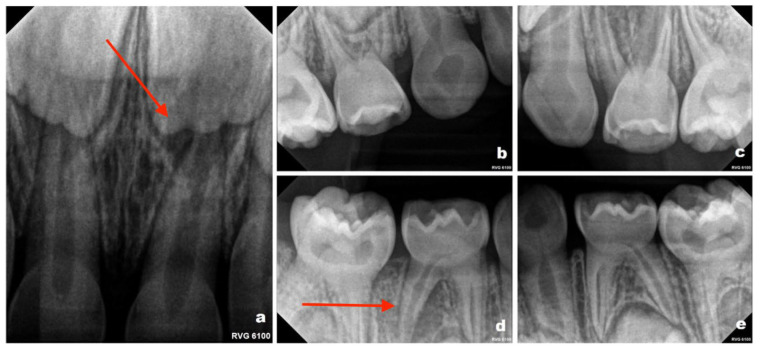
Periapical radiographs obtained in September 2022 (patient aged 3 years): (**a**) maxillary anterior region showing a periapical radiolucency associated with tooth 61 (Please note the area indicated by the red arrow in the figure); (**b**) upper right quadrant; (**c**) upper left quadrant; (**d**) lower right quadrant, demonstrating periapical changes at the distal root of tooth 84 (Please note the area indicated by the red arrow in the figure); (**e**) lower left quadrant.

Tooth numbering in this report follows the Fédération Dentaire Internationale (FDI) two-digit notation system. Periapical radiographic evaluation (Figure 2a–e) revealed periapical changes associated with teeth 61 and 84. All first primary molars demonstrated obliteration of the pulp chambers and root canals, a radiographic feature consistent with DI. Teeth 53 and 73 exhibited marked narrowing of the pulp space in the cervical region. Tooth 83 could not be fully evaluated because it was not included within the radiographic field. Panoramic radiography was not performed during the course of treatment. Imaging was limited to periapical radiographs, which were considered sufficient for localized assessment of the affected teeth. Given the patient’s young age and variable cooperation, as well as the aim to minimize radiation exposure, more extensive imaging was not routinely indicated at that stage. Evaluation of the developing permanent dentition was therefore not performed radiographically during the early follow-up period. Radiographic monitoring was planned at approximately 6-month intervals, based on the patient’s high caries risk and susceptibility to dental pathology; however, the timing of imaging was also adjusted according to the child’s level of cooperation and clinical circumstances at each visit.

In the absence of clinical symptoms and considering the patient’s young age, limited cooperation, and the absence of extensive structural breakdown at that stage, a conservative, risk-based approach was adopted. Teeth 61 and 84 were placed under close clinical and radiographic observation, with the understanding that radiographic progression—rather than symptom onset alone—would guide intervention. Although full-coverage restorations such as stainless steel crowns are frequently recommended in DI to protect tooth structure and maintain occlusal stability, their placement in very young or minimally affected teeth may be limited by behavioral factors, tooth morphology, and the absence of clear evidence defining optimal timing. Therefore, in this case, a preventive and minimally invasive approach was initially prioritized, with the option of escalation to full-coverage restorations if structural breakdown or functional compromise became evident.

The child demonstrated variable cooperation during dental visits but was generally cooperative enough to allow treatment under local anesthesia. Pharmacological approaches, including inhalation sedation and general anesthesia, were considered but were not deemed necessary, as the required procedures were of limited duration and could be completed in a conventional clinical setting.

As part of preventive management aimed at reducing caries risk and limiting structural deterioration, occlusal sealants were placed on teeth 55, 65, 75, and 85. At that time, all erupted primary teeth exhibited discoloration except the most recently erupted molars. Although stainless steel crowns are commonly recommended for posterior teeth in DI, they were not used in this case due to the patient’s limited cooperation and the expectation that such treatment would require general anesthesia, which was not justified at that stage.

During this period, the parents reported significant psychoemotional stress experienced by the child in kindergarten due to peer reactions to tooth color. Following a shared decision-making process, direct composite veneers were planned for the four maxillary primary incisors to address esthetic concerns and support psychosocial well-being. This approach was selected with the understanding that veneers improve surface appearance but do not modify the underlying dentin defect.

Direct composite veneers were chosen as a minimally invasive option that preserves tooth structure while providing acceptable short-term esthetic outcomes. However, it was recognized that bonding to dentin affected by DI may be less predictable and that such restorations are not expected to prevent pulpal or periapical pathology [21]. Alternative options, including strip crowns and full-coverage restorations, were considered but were not used due to material availability, the patient’s age, and cooperation level. A conservative approach was therefore preferred to preserve remaining tooth structure.

Non-pharmacological behavior management techniques (e.g., tell–show–do and positive reinforcement) were applied throughout the veneer placement procedures to facilitate cooperation and allow treatment to be completed under local anesthesia.

Prior to veneer placement, the labial surfaces were minimally prepared. In this case, “minimal preparation” consisted of superficial enamel roughening rather than measurable reduction, to enhance micromechanical retention while preserving enamel and avoiding unnecessary exposure of structurally compromised dentin.

Veneers were placed on teeth 51, 52, 61, and 62 in two sessions in January and February 2023. Treatment was staged to accommodate the child’s limited tolerance for prolonged procedures. All restorations were performed under local anesthesia. Salivary isolation was achieved using cotton rolls without rubber dam placement due to behavioral considerations. Composite materials were applied using a standard adhesive protocol with both flowable and packable composites (Kerr OptiBond Solo Plus, Megafill MH A1, Megafill Flow A1).

**Figure 3:**
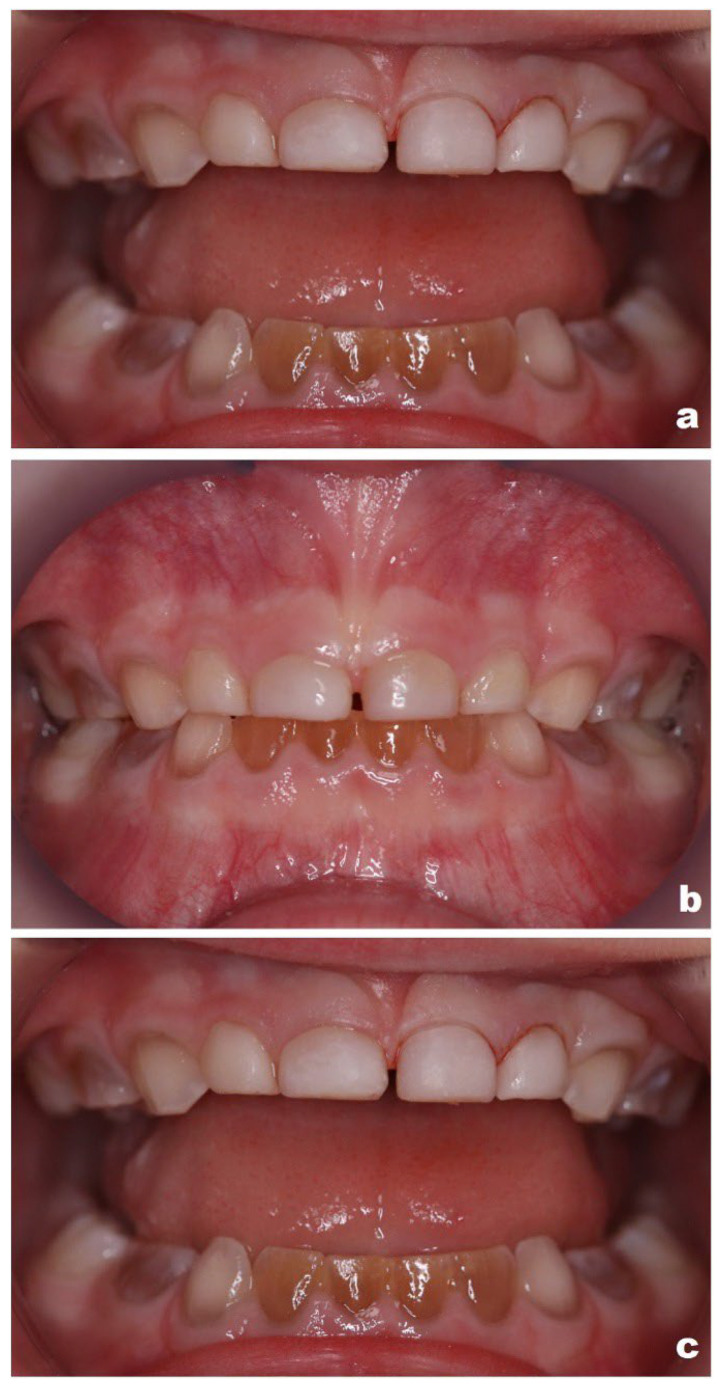
Intraoral photographs of direct composite veneers on the maxillary anterior teeth (patient aged 4–5 years): (**a**) immediately after placement of the final two veneers during the second visit (patient aged 4 years); (**b**) six months postoperatively; (**c**) one year postoperatively (patient aged 5 years).

Follow-up examinations in January and August 2024 demonstrated clinically stable restorations, with improvement in psychosocial well-being based on parental reports, including reduced peer-related distress (Figure 3).

**Figure 4:**
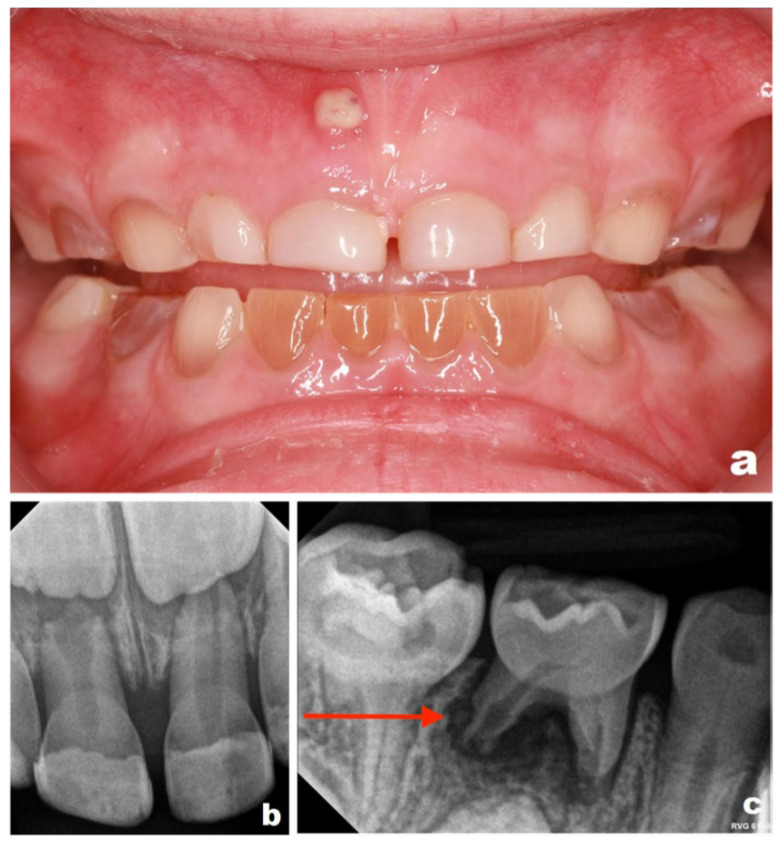
Intraoral photographs and periapical radiographs obtained in January 2025 (patient aged 5 years): (**a**) clinical photograph showing a sinus tract adjacent to tooth 51; (**b**) periapical radiograph of tooth 51 demonstrating periapical inflammation and pathological widening of the root canal; (**c**) periapical radiograph of tooth 84 revealing periapical inflammation (Please note the area indicated by the red arrow in the figure).

At a scheduled follow-up visit in January 2025, clinical examination revealed a sinus tract in the region of tooth 51. Periapical radiographs demonstrated pathological root resorption associated with tooth 84 and periapical inflammation related to tooth 51. Intraoral photographs were obtained to document these findings (Figure 4). While these findings indicate disease progression, it cannot be determined whether earlier restorative intervention would have altered the clinical course.

**Figure 5:**
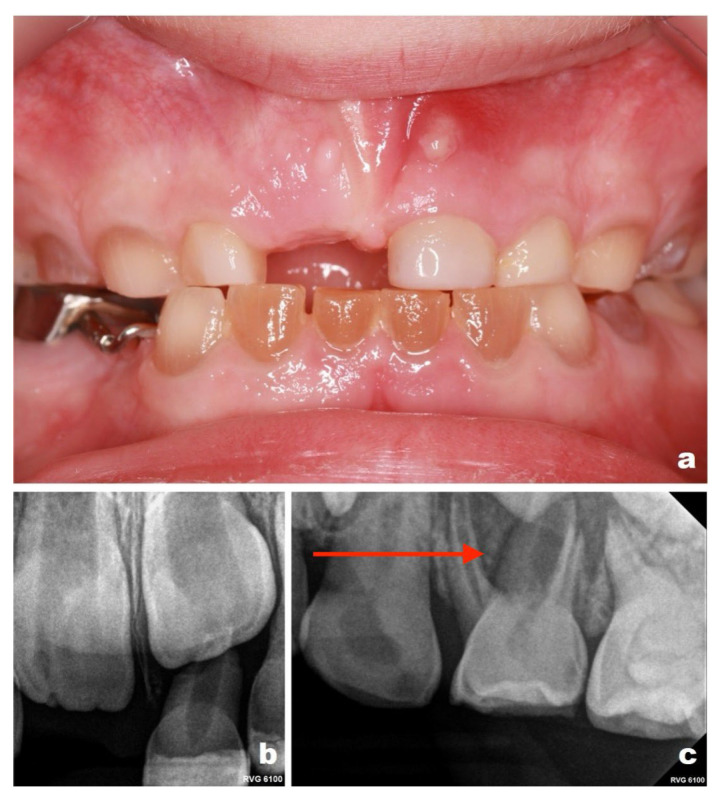
Intraoral photographs and periapical radiographs obtained in September 2025 (patient aged 6 years): (**a**) clinical photograph showing a sinus tract adjacent to tooth 61; (**b**) periapical radiograph of tooth 61 demonstrating root resorption and proximity to the permanent successor; (**c**) periapical radiograph of tooth 64 revealing periapical inflammation (Please note the area indicated by the red arrow in the figure).

Given the radiographic evidence of infection, extractions of teeth 84 and 51 were performed to eliminate active pathology. A space maintainer was placed in the region of the extracted tooth 84 to preserve arch length. At a subsequent follow-up visit in September 2025, periapical radiographs revealed inflammatory changes associated with tooth 64 and a sinus tract in the region of tooth 61 (Figure 5). As tooth 64 remained clinically asymptomatic and close to exfoliation, extraction was deferred until eruption of tooth 26. In contrast, tooth 61 was extracted to control infection. These decisions were based on a combination of radiographic findings, clinical status, and expected timing of exfoliation, rather than symptoms alone.

Throughout the course of care, including at times when radiographic pathology was evident, the child and parents consistently reported no pain, swelling, or functional limitations. Notably, no subjective complaints were associated with teeth 84, 64, 51, or 61. This finding highlights the well-recognized discrepancy between clinical symptoms and underlying disease activity in DI and supports the use of structured radiographic surveillance as a key component of management.

To improve clarity of the longitudinal follow-up, a summary of clinical visits, findings, and interventions is provided in a table format (Table 1).

## 3. Discussion

Epidemiological data on DI are often reported in association with OI with prevalence rates ranging from 19% to over 60% depending on population characteristics and OI subtype distribution [22]. However, such data have limited relevance to isolated DI, as in the present case, and should be interpreted cautiously. To date, no epidemiological data are available for Latvia.

In the present patient, the clinical and radiographic findings, together with the absence of systemic features, are most consistent with a clinical presentation suggestive of dentinogenesis imperfecta type II. However, in the absence of genetic confirmation and a detailed pedigree analysis, this classification should be interpreted as a clinically supported impression rather than a definitive diagnosis. Osteogenesis imperfecta was not clinically supported, as there were no reported signs of bone fragility, growth abnormalities, or other systemic manifestations during the observation period.

DI presents significant clinical challenges due to the structural fragility of affected dentin, limiting the predictability of restorative procedures [8]. In this case, management decisions were guided by disease severity, patient age, cooperation, and psychosocial factors. The reported peer-related distress influenced treatment planning, consistent with evidence that caregiver perspectives play a key role in decision-making [10,23].

Despite general agreement on the importance of early intervention, there is still no universally accepted, evidence-based guideline for managing DI in the primary dentition [24]. Consequently, clinical practice is largely guided by clinician experience and individual case factors. Existing reports consistently describe an increased risk of structural breakdown and caries progression, including enamel–dentin interface failure and increased plaque retention [25,26].

Preventive care, including sealant placement and regular monitoring, formed the basis of initial management. Although stainless steel crowns are commonly recommended to protect tooth structure and maintain occlusal stability [8,27], they were not used due to the patient’s age, limited cooperation, and the aim to avoid treatment requiring general anesthesia. This highlights the need to individualize treatment timing rather than applying standardized protocols.

Direct composite veneers were placed primarily for psychosocial benefit. While they provided short-term esthetic improvement, they did not address the underlying dentin defect. The subsequent development of pathology in anterior teeth reinforces that such restorations should not be considered protective against pulpal or periapical complications [28].

A key observation in this case is the development of radiographic pathology in the absence of clinical symptoms. Teeth 64 and 84 exhibited pathological changes despite the absence of pain or functional complaints. This finding has direct clinical implications, indicating that symptom-based assessment alone is insufficient in DI and that regular radiographic monitoring is essential. Importantly, causality cannot be established, and it remains unclear whether earlier intervention, including full-coverage restorations, would have altered the clinical course.

Comparison with published reports demonstrates variability in management strategies. Some authors advocate early placement of stainless steel crowns [14,29], while others report the use of alternative materials such as nanoceramic crowns [30]. In syndromic cases associated with OI, treatment planning is further influenced by systemic factors [31]. Earlier reports have also described conservative and combined pedodontic–orthodontic approaches, including the use of crowns and space maintainers to preserve arch integrity [32,33]. However, most reports lack long-term follow-up, limiting conclusions regarding treatment effectiveness. In contrast, the present case contributes longitudinal data and highlights that disease progression may occur despite preventive and restorative measures.

General anesthesia is sometimes required in young or uncooperative patients [34]; however, in this case, treatment was successfully completed under local anesthesia using a staged approach. This suggests that, in selected patients, comprehensive care may be achieved without general anesthesia when appropriately adapted.

Early diagnosis and conservative management remain key principles in DI care [35]. Treatment planning should consider the stage of dentition, with primary teeth requiring preservation of function and space, while permanent dentition demands more durable solutions [31]. Referral for genetic evaluation may be appropriate when systemic involvement is suspected [19,20,31], although in this case genetic consultation was recommended but not pursued.

Endodontic treatment in DI is challenging due to pulp canal obliteration and abnormal dentin structure and is often associated with poor long-term outcomes [16,36]. Consequently, extraction may be required when pathology develops.

The principal strength of this report lies in its longitudinal clinical and radiographic documentation, demonstrating progression of pathology despite minimal symptoms. However, as a single case, the findings are not generalizable, and the absence of histological confirmation limits interpretation. Rather than establishing causality, this case provides clinically relevant insight into the discrepancy between symptoms and disease activity, supporting risk-based monitoring and individualized management.

## 4. Conclusions

This case highlights the importance of early and ongoing surveillance in children with *dentinogenesis imperfecta*, as pulpal and periradicular pathology may develop in the absence of clinical symptoms. In the present patient, radiographic changes were identified despite minimal subjective complaints, supporting the need for routine radiographic monitoring as part of follow-up.

Management should be individualized, taking into account patient age, cooperation, and clinical presentation. In this case, a conservative and staged approach enabled both preventive care and esthetic rehabilitation. Although findings from a single case cannot be generalized, this report underscores the discrepancy between clinical symptoms and disease activity and supports risk-based monitoring strategies.

## Figures and Tables

**Table 1:** Summary of clinical visits.

Date	Age	Key Findings	Radiographic Findings	Intervention	Outcome
May 2020	1.5 y	Generalized tooth discoloration; suspected DI	Not performed	Initial assessment	Enrolled in recall program
Dec 2020–Aug 2022	2–3 y	Stable condition; intermittent sensitivity	Not performed	Routine follow-up	No acute symptoms
Sept 2022	3 y	Asymptomatic	Periapical changes (61, 84); pulp obliteration molars	Preventive plan; monitoring initiated	Conservative approach adopted
Sept 2022	3 y	Full dentition discoloration	Same as above	Sealants on 55, 65, 75, 85	Caries prevention
Jan–Feb 2023	3.5 y	Psychosocial distress (esthetics)	Not repeated	Composite veneers (51, 52, 61, 62)	Improved esthetics and well-being
Jan & Aug 2024	4–5 y	Stable restorations	Not performed	Follow-up	No complications reported
Jan 2025	5.5 y	Sinus tract (51)	Pathology: 51, 84	Extraction (51, 84); space maintainer	Infection controlled
Sept 2025	6 y	Sinus tract (61); asymptomatic 64	Pathology: 61, 64	Extraction (61); monitoring (64)	Ongoing management

## Data Availability

The data presented in this study are available on request from the corresponding author. The data are not publicly available due to patient data protection law and confidentiality.

## Abbreviations

The following abbreviations are used in this manuscript:

DI	*Dentinogenesis Imperfecta*
OI	*Osteogenesis Imperfecta *
AI	*Amelogenesis Imperfecta *
